# Parkinson's Disease Database in the Middle East, North Africa, and South Asia Countries

**DOI:** 10.3389/ijph.2025.1608016

**Published:** 2025-06-02

**Authors:** Hanan Khalil, Mujahed Shraim, Bayan Jaradat, Riham Hussein Ibrahim, Walaa A. Kamel, Shazma Khan, Mayis Aldughmi, Hanan Amer, Ahmed Al-Qassabi, Alham Al-Sharman, Ahsan Habib, Aljoharah Alakkas, Mehri Salari, Shaimaa I. El-Jaafary, Junaid Siddiqui, Zakiyah Aldaajani, Duha M. Al-Shorafat, Khalid Elsalem, Asma Alhamid, Tareq M. Mohammad, Malak Nasser Alkahtani, Najd Khalid Alrumaihi, Fatimah AlHawiti, Malak Ruwaished Albadrani, Iman Zaynab Bajwa, Ayah Khrisat, Ahmed Dahshan, Asmaa Sabbah, Nesma Mounir Abd Algaber, Hatem Samir Shehata, Sarah Sherif Abdo, Shaimaa A. Elaidy, Nesma A. M. Ghonimi, Amina Nasri, Yasmine Mrad, Youssef Abida Abida, Riadh Gouider, Mahmood Khalifa Al Hinai, Badriya Masoud Alhosni, Mohammed Farghal, Heba Shinawi, Omar Alsinaidi, Shatha Al Sariri, Junaid Ahmed, Naeemullah Bullo, Rida Younis, A. B. S. M. Sirajul Haque, Nayeem Anwar, Ranjit Kumar Ghosh, Jahirul Hoque Chowdhury, Abu Nayeem, Mohammad Kafil Uddin, Mohammad Ibrahim Khalil, Md. Rashedul Islam, Salma Mohamed Ragab, Mahmoud Farid, Fatima Jamali, Akhtar Sherin, Wajeeha Bokhari, Sohail Adnan, Aly Hassan, Qamar un Nisa, Irfan Hashmat, Warda Fatmi, Jawad A. Bajwa

**Affiliations:** ^1^ Department of Rehabilitation Sciences, College of Health Sciences, QU Health, Qatar University, Doha, Qatar; ^2^ Faculty of Applied Medical Sciences, Department of Rehabilitation Sciences, Jordan University of Science and Technology, Irbid, Jordan; ^3^ Neurology Department, Faculty of Medicine, Beni-Suef University, Beni-Suef, Egypt; ^4^ Neurology Department, Ibn Sina Hospital, Kuwait City, Kuwait; ^5^ Department of Neurology, Dr. Ziauddin University Hospital, Karachi, Pakistan; ^6^ Department of Physiotherapy, School of Rehabilitation Sciences, University of Jordan, Amman, Jordan; ^7^ Neurology Department, Faculty of Medicine, Cairo University, Giza, Egypt; ^8^ Neurology Unit, Department of Medicine, Sultan Qaboos University Hospital, Medical University City, Muscat, Oman; ^9^ Physiotherapy Department, Faculty of Health Sciences, University of Sharjah, Sharjah, United Arab Emirates; ^10^ Department of Neurology, Bangabandhu Sheikh Mujib Medical University, Dhaka, Bangladesh; ^11^ Department of Neurology, National Neuroscience Institute, King Fahd Medical City, Riyadh, Saudi Arabia; ^12^ Men's Health and Reproductive Health Research Center, Shahid Beheshti University of Medical Sciences, Tehran, Iran; ^13^ Center for Neurological Restoration, The Neurological Institute, Cleveland Clinic, Cleveland, OH, United States; ^14^ Neuroscience Department, King Fahad Military Medical Complex, Dhahran, Saudi Arabia; ^15^ Neurology Department, Jordan University of Science and Technology, Irbid, Jordan; ^16^ Medical College, Aga Khan University, Karachi, Pakistan; ^17^ Neurology Department, Faculty of Medicine, Zagazig University, Zagazig, Egypt; ^18^ Department of Neurology, LR 18SP03 and Clinical Investigation Center (CIC), Clinical Investigation Centre Neurosciences and Mental Health, Razi University Hospital, Tunis, Tunisia; ^19^ Faculty of Medicine of Tunis, University of Tunis El Manar, Tunis, Tunisia; ^20^ Neurology Department, Directorate General of Khoula Hospital, Muscat, Oman; ^21^ Neurology Division, Al-Adan Hospital, Kuwait City, Kuwait; ^22^ Jinnah Post Graduate Medical Centre, Karachi, Sindh, Pakistan; ^23^ Department of Neurology, Salmaniya Medical Complex, Kingdom of Bahrain; ^24^ Department of Neurology, National Institute of Neurosciences and Hospital, Dhaka, Bangladesh; ^25^ Department of Neurology, Shaheed Suhrawardy Medical College and Hospital, Dhaka, Bangladesh; ^26^ Bangladesh Institute of Research and Rehabilitation for Diabetes Endocrine and Metabolic Disorders (BIRDEM), Dhaka, Bangladesh; ^27^ Neurology Department, Faculty of Medicine, Kafrelsheikh University, Kafr El-Sheikh, Egypt; ^28^ Cell Therapy Center, the University of Jordan, Amman, Jordan; ^29^ Khyber Medical University Institute of Medical Sciences, Kohat Development Authority Hospital KDA, Kohat, Pakistan; ^30^ Neurology Medical Clinic-Medical Affairs, Tawam Hospital, Al Ain, United Arab Emirates; ^31^ Department of Neurology, Dr. Ruth Pfau Civil Hospital, Dow Medical College, DUHS, Karachi, Pakistan

**Keywords:** quality of life, patient care, neurodegenerative diseases, Parkinson’s disease, multicenter database

## Abstract

**Objectives:**

This study aims to establish a multicenter database to evaluate Parkinson’s disease in the MENASA region in the context of expert care.

**Methods:**

The CGD-PD consortium includes 20 institutes from 9 MENASA countries. The database collects comprehensive data from PD patients.

**Results:**

Initial data from participating sites showed significant heterogeneity in patient demographics, clinical characteristics, and healthcare management within the MENASA area. Descriptive analyses will include patient demographics and treatment methods, while multilevel regression models will explore correlations across care levels, environmental factors, and health outcomes. The results are anticipated to reveal region-specific patterns and gaps in the management of Parkinson’s disease.

**Conclusion:**

The CGD-PD database will be instrumental in addressing the gap in PD research in the MENASA region, ultimately improving the quality of life for PD patients.

## Introduction

Parkinson’s disease (PD) is a neurodegenerative disorder primarily associated with motor features of tremor, rigidity, bradykinesia, and postural instability. Globally, PD has increased significantly since the 1990s [1], and it is currently the second most common neurodegenerative disorder after Alzheimer’s disease, with a worldwide prevalence of 8.5–10.8 million [1–3].

Across the Middle East, North Africa, and South Asia (MENASA), many countries have experienced an improvement in their socioeconomic status. The combination of increasing longevity and lower birth rates common with increasing wealth has contributed to an aging population and increasing prevalence of the diseases of aging. For example, when examining worldwide PD trends between 1990 and 2019, the largest increase in incidence was seen in Qatar (796.51%) and the United Arab Emirates (854.71%) [4].

The exact cause of PD is not completely understood; however, it may depend on environmental factors affecting a genetically susceptible individual [2, 5]. Caffeine, pesticide exposure, age, and family history are all factors associated with the development of PD [6]. The prevalence of PD between 65 and 69 years of age is 0.5%–1%. This rises to 1%–3% in those older than 80 [7, 8]. In one study, having a family history of PD was the strongest factor in the development of PD [6]. Caffeine was linked with a reduced risk of PD in several human and animal studies [6, 9–11]. The factors stated above differ across culturally. For example, the average annual total caffeine volume sales *per capita* were highest in North America, at 348 L *per capita*, and lowest in Africa, at 90 L *per capita* [12]. In addition, a study of daily caffeine intake patterns in the US found that ethnicity was one of the variables most strongly associated with intake. The difference remained significant even when employment status and weekly work hours were adjusted for [13]. Regarding genetic susceptibility, the MENASA region is characterized by high rates of consanguineous marriage, leading to a relatively high prevalence of genetic disorders. Thus, the epidemiology of PD in the MENSA region can shed light into the pathogenesis of PD. Additionally, understanding the regional necessities required to treat PD is important to prioritize research and public health policies. Overall, there is a significant lack of research on PD in the MENSA region. For this reason, the Consortium for Global Diversity in PD (CDG-PD) was established in 2020. CDG-PD comprises a network of PD experts from the MENASA region. The mission of the CDG-PD is “to improve the quality of care for people with PD through research, education and outreach.”

The CGD-PD database will provide the first large-scale, region-specific analysis of PD experiences, treatments, and care models in MENASA. The insights gained will help improve patient quality of life, enhance caregiver support, and guide healthcare policies to provide better and more equitable PD care.

In this paper, we present the study protocol of a multicenter database of patients with PD in the MENASA region that was created as a result of the CDG-PD initiative. The aims of establishing this database are to [1]: describe the clinical characteristics of PD patients in the MENASA region [2], explore the current treatments and level of care for patients with PD across centers in the MENASA region [3], assess the socioeconomic and financial burden of PD in the MENASA region [4], explore the relationship between PD-related treatments and levels of care with patient-perceived current health status, and [5] explore the relationship between environmental exposure and clinical characteristics of PD patients in the MENASA region.

The development of this consortium and regional database is the first step towards helping healthcare professionals, researchers and health policymakers to better understand PD and to improve the care of people with PD in MENASA. The development of the database will also help in the future in identifying patients who may wish to participate in clinical trials, facilitate efforts for quality improvement required to enhance the quality of life of people with PD, and generate recommendations for patient education, healthcare professionals and policymakers.

## Methods

### Establishing the Consortium

Consortium included participating centers from the MENASA region (n = 20). This involved 9 countries including Saudi Arabia, Kuwait, Oman, Iran, Jordan, Egypt, Tunisia, Bangladesh, and Pakistan. The selection of participating centers was not based on strict inclusion or exclusion criteria. Instead, we sent invitations to multiple centers and hospitals, and those that responded and met essential requirements were included in the study.

The primary considerations for participation were the presence of a PD treatment center or hospital that provided care for PD patients to ensure relevant data collection and the ability to obtain Institutional Ethical Approval to maintain ethical standards.

There were no predefined exclusion criteria; however, institutions that were unable to obtain IRB approval were not included. This approach ensured broad participation from diverse regions within MENASA, allowing for a comprehensive and representative dataset on PD care and patient experiences. Details of the participating centers are included in Table 1.

**TABLE 1:** Participating Centers in the Consortium. Parkinson's Disease Database in the Middle East, North Africa and South Asia Countries, 2024.

Country	City	Center(s)
Bangladesh	Dhaka	Bangabandhu Sheikh Mujib Medical University (BSMMU)
		National Institute of Neurosciences (NINS)
		Shaheed Shuhrawardy Medical College
		Bangladesh Institute of Research and Rehabilitation in Diabetes, Endocrine and Metabolic Disorders (BIRDEM)
Egypt	Cairo	Kasr Alainy University Hospital, Cairo University
	Kafr El Sheikh	Kafrelshaikh University Hospital
	Zagazig	Zagazig University
Iran	Tehran	Shahid Beheshti University of Medical Science- Shohada-e-Tajrish Hospital
Jordan	Amman	University of Jordan
	Irbid	Jordan University of Science and Technology
Kuwait	Kuwait City	Ibn-Sina Hospital
Oman	Muscat	Sultan Qaboos University
		Khoula Hospital
Pakistan	Karachi	Jinnah Postgraduate Medical Center
		Ziauddin University Hospital
		Dow University of Health Sciences
	Kohat	Khyber Medical University Institute of medical sciences, KDA Teaching hospital
Saudi Arabia	Dhahran	King Fahad Military Medical Complex
	Riyadh	King Fahad Medical City
Tunisia	Tunis	Razi University

### Project Governance

The development and progress of the database was overseen by Project Governance (PG). The (PG) involves at least one representative from each center. The (PG) meet at least once a month by audio conference. The (PG) acts as a data monitoring and ethics committee. The PSC also provides overall supervision of the study and ensures that it is being conducted in accordance with the principles of Good Clinical Practice. The (PG) also agreed the project protocol and any protocol changes and provides advice on all aspects of the project. The (PG) oversees the progress of the project, including the recruitment, data completeness, and ensures that there are no major deviations from the study protocol.

### Participants

Any person with the diagnosis of idiopathic PD who receives medical care for PD at any of the participating centers is eligible to participate. There is no exclusion for participants based on age, severity of the disease or presence of cognitive impairments. All participants will give written informed consent approved from the Institutional Research Committees (IRB) of the participating centers; each site obtained their IRB approval separately.

### The Database

The Database contains information related to nine pillars [1]: demographic data including age, gender, race, ethnicity, living arrangements, and educational level [2], family history of PD [3], environmental exposures including residential history, heavy metal use and others [4], clinical characteristics of the disease including motor and non-motor symptoms as well as co-morbid conditions, and number of hospitalization and emergency care admissions in the last year [5], current treatments including pharmacological, surgical and other models of care such as physiotherapy, occupational therapy and speech and language therapy [6], availability of care and care giver [7], physicians and patients’ perceived change of patients’ current health comparing to the last medical visit [8], Patient’ engagement in regular physical activity [9], financial burden of the disease. Some of this data is collected using tests and questionnaire instruments that are currently in regular use in clinical practice. In addition, a questionnaire was specifically developed for the purposes of this project to collect relevant and needed information to the MENASA region.

Overall, data to include on the database was decided by constructing a conceptual framework based on the available literature, and discussion with experts in that field. Hence, this helped in clearly defining the database objectives and the relevant information that needs to be collected. Accordingly, a questionnaire was created which was reviewed by the study consortium and experts in the field (See Supplementary Appendix SA1 for the full version of the questionnaire). In building the questionnaire, attention was given to simple wording of the questions, logical ordering of the questions, and time needed to collect all needed data.

### Translation of the Developed Questionnaire Into Different Languages

The questionnaire was created in English and then was translated into 4 languages (Arabic, Persian, Urdu, and Bangla); these languages represent the main languages spoken in all the participating centers. To ensure the accuracy of the translated versions, a back-translation method was used in which at least two people who were proficient in both English and the translated language had compared the original questionnaire with the back-translated English questionnaire for any discrepancy. Comments were discussed with the (PG) to reach a final consensus and final version of the questionnaire in the different languages.

The primary consideration in the development and translation of the questionnaire was guaranteeing cultural appropriateness across the MENASA region. We conducted detailed discussions before obtaining the questionnaire to ensure its integration and accessibility. The questionnaire was developed with simple, forward language, and interviewers were trained to provide clarifications to accommodate any differences. The administration of the questionnaire accommodated cultural differences such as religion, social, educational, literacy, and gender differences.

### Piloting of the Developed Questionnaire

The pre-final version of the questionnaire (in the 4 languages) was piloted. A small number of respondents were selected from several centers to test if the questions are best worded and placed, if any questions should be added or eliminated, and whether the instructions are adequate. Based on feedback and discussion with the project consortium, a final version of the questionnaire was created.

### Data Management

To ensure the quality of the data collected, training material on data collection and data entry was provided by the (PG) to all investigators involved in the project. In addition, data entry was checked by an independent researcher and one to one debriefing session was conducted to all investigators who were involved in data collection and data entry from all sites.

Data will be collected on paper data forms by the principal investigators from each participating center who manage the patients. These investigators will ask the relevant questions and extract the necessary data from the patients’ medical records to ensure accurate and comprehensive responses. Only de-identified data will be shared with researchers at Qatar University and Jordan University of Science and Technology for screening and verification purposes. In the future, data collection may transition to secure, encrypted online systems (including web-based platforms) at all centers. Throughout both phases, the database will be housed in a secure facility that complies with legal standards for both physical and electronic data security. Data transmission will be encrypted and sent through secure channels, or via registered mail for any paper-based records.

### Data Analysis

The variables will be summarized using descriptive statistics, with means and standard deviations or medians and interquartile ranges for continuous variables, and frequencies and percentages for categorical variables, as appropriate. Additionally, descriptive statistics will be used to address objectives 1–3. Moreover, multilevel mixed models will be employed to address objectives 4-5.

## Results

Table 2 provides a comprehensive overview of patient characteristics to be reported using descriptive statistics upon completion of data analysis. This includes demographic and socioeconomic factors; clinical and behavioral characteristics; PD-related symptom severity, surgical history and medication use; residential and environmental exposures; health coverage; and financial toxicity-related assessments. Each variable is detailed alongside its corresponding data format (Table 2). Multilevel ordinal logistic regression will be employed to assess the relationships between PD-related treatments and levels of care (including PD-related surgery, medication use, and various therapies) with patient-perceived current health status. Crude and adjusted odds ratios (ORs), along with 95% confidence intervals (CIs), will be reported and presented in a table as measures of these associations.

**TABLE 2:** Summary of Patient Characteristics and Variables Parkinson's Disease Database in the Middle East, North Africa and South Asia Countries, 2024.

Characteristics	Description/Categories	Format
Demographic and Socioeconomic		
Age (years)	Mean ± SD/Median ((nterquartile range (IQR))	Continuous
Sex	Male, Female	Categorical
Race	White/Caucasian, Black, Brown, Yellow, Multi-racial, Others	Categorical
Ethnicity	Middle Eastern Arab, North African Arab, Berber, Persian, Kurdish, Armenian, Turkish, Bengali, Punjabi, Pashtun, Sindhi, Balochi, Indo-Aryan, Jewish, African (None-North African), Nubians, Indian, Others	Categorical
Marital status	Single or never married, Married or domestic partner, Divorced or separated, Widowed	Categorical
Education level	Less than high school, high school, post-high school education or associate’s degree, bachelor’s degree, graduate degree (master’s, professional, or doctoral)	Categorical
Employment status	Employed full-time, Employed part-time, Not employed, Retired	Categorical
Living arrangements	Living with someone who can assist with daily activities if needed (Yes, No)	Categorical
Clinical		
Body Mass Index (kg/m^2^)	Mean ± SD/Median ((nterquartile range (IQR))	Continuous
Physical activity intensity (hours/week): Light-intensity (e.g., walking at a leisurely pace, household or yard chores, seated exercises); moderate-intensity (e.g., brisk walking, walking on hills, dancing, Tai Chi, yoga, Pilates, arm or leg cycling, pool aerobics); vigorous-intensity exercise (e.g., stair climbing, swimming laps, weightlifting)	Mean ± SD/Median ((nterquartile range (IQR))	Continuous
Coffee or tea intake status	Yes, No Types: Latte/Cappuccino (coffee with milk), Americano/Espresso, Arabic coffee, Black tea, Green tea, Black tea with milk, Others	Categorical
Intensity of coffee or tea intake (day)	<2 cups per week, 2–6 cups per week, 1–2 cups per day, 3–5 cups per day, ≥6 cups per day	Categorical
Soda intake status	Yes, No	Categorical
Intensity of soda intake	<1 can per week, 2–3 cans per week, 1–2 cans per day, 3–6 cans per day, >6 cans per day	Categorical
History of cigarette smoking	Yes, No	Categorical
Intensity of cigarette smoking (per day)	≤½ pack, ≥½ pack to <1 pack, ≥1 pack to <2 packs, ≥2 packs	Categorical
Duration of cigarette smoking (years)	Mean ± SD/Median (IQR)	Continuous
History of use for each of the followings tobacco forms: Chewable tobacco, Huqqa, Sheesha, Other forms	Yes, No	Categorical
History of drugs use: Amphetamine, Methamphetamine, Cocaine, or Heroin	Yes, No, Prefer not to answer	Categorical
Family history of PD	Yes, No, Don’t Know	Categorical
History of head injury affecting cognition	Yes, No, Don’t Know	Categorical
Standing unaided status	Yes, No	Categorical
Attendance of activities outside the home unaccompanied	Yes, No	Categorical
Attendance of any support groups	In-Person, Online, Both, Other, No	Categorical
PD symptoms		
Duration since PD symptom onset (years)	Mean ± SD/Median ((nterquartile range (IQR))	Continuous
Duration since PD diagnosis (years)	Mean ± SD/Median ((nterquartile range (IQR))	Continuous
Type of initial symptoms of PD	Motor, Non-motor, Both	Categorical
Laterality of initial symptoms of PD	Right, Left, Bilateral	Categorical
History of rest tremors since diagnosis of PD	Yes, No	Categorical
History of dyskinesia (last 30 days) and its impact activities	Yes, it limits activities; Yes, it does not limit activities; No	Categorical
Severity of PD symptoms	Stage 0: No signs of disease; Stage 1.0: Symptoms are very mild, unilateral involvement only; Stage 1.5: Unilateral and axial involvement; Stage 2: Bilateral involvement without impairment of balance; Stage 2.5: Mild bilateral disease with recovery on pull test; Stage 3: Mild to moderate bilateral disease, some postural instability, physically independent; Stage 4: Severe disability, still able to walk or stand unassisted; Stage 5: Wheelchair bound or bedridden unless aided	Categorical
History of non-motor symptoms at PD onset: Depression, Anxiety, Mild cognitive impairment, Apathy, Fatigue, Insomnia, Restless leg syndrome, Rapid eye movement disorders, Daytime sleepiness, Constipation, Urinary tract symptoms, Pain or muscle cramps, Orthostatic hypotension or dizziness when standing, Anosmia	Yes, No	Categorical
History of PD-related conditions: Visual hallucinations, memory decline, freezing episodes, sleep disturbances, sleep behavior disorder, rapid eye movement sleep behavior disorder, Dopamine Dysregulation Syndrome	Yes, No	Categorical
History of comorbid conditions: Hypertension, Heart disease, Lung disease, Cancer, Diabetes, Stomach ulcer or disease, Liver disease, Kidney disease, Depression, Psychosis, Osteoarthritis or degenerative arthritis, Rheumatoid arthritis, Back pain, Benign prostate hypertrophy	Yes, No	Categorical
Frequency of falls (last 3 months)	None, Rarely, Monthly, Weekly, Daily	Categorical
Number of emergency room visits (last 12 months)	Mean ± SD/Median ((nterquartile range (IQR))	Count
Number of hospital admissions (last 12 months)	Mean ± SD/Median ((nterquartile range (IQR))	Count
Compliance with PD medications	Yes, No	Categorical
Current use of therapies: Physical therapy, Occupational therapy, Speech language pathologist for communication or swallowing, Dietitian, Psychologist, Psychiatrist	Treatment plan before current visit: No, Yes in the last 3 months, Yes 4–12 months. Treatment plan after current visit: To be started, To be continued, Stopped or not needed.	Categorical
Institutional exercise program (hours/week)	Mean ± SD/Median ((nterquartile range (IQR))	Continuous
PD-related surgical history		
History of deep brain stimulation surgery	Yes, No	Categorical
Laterality of deep brain stimulation surgery	Unilateral, Bilateral	Categorical
Targets of deep brain stimulation surgery	Suubthalamic neucli (STN), The pedunculopontine nucleus (PPN), Globus Pallidus Internus (Gpi), Others	Categorical
History of lesion surgery	Yes, No	Categorical
History of infusion pump use	Yes, No	Categorical
Type of infusion pump	LCIG, Apomorphine	Categorical
Duration since infusion pump use (years)	Mean ± SD/Median ((nterquartile range (IQR))	Continuous
PD-related medication use		
Amantadine, Trihexyphenidyl: Procyclidine Hydrochloride, Carbidopa/levodopa-Immediate release, Carbidopa/levodopa-controlled release, Entacapone, Carbidopa/levodopa-extended release, Carbidopa/levodopa/entacapone, Levodopa inhalation powder, Selegiline, Safinamide, Rasagiline, Pramipexole Immediate release, Pramipexole-extended release, Ropinirole-immediate release, Ropinirole-extended release, Rotigotine transdermal patch, Apomorphine-slow release wafer, Apomorphine injection, Bromocriptine, Istradefylline, Quetiapine, Quetiapine-extended release, Clozapine, Pimavanserin, Rivastigmine: Exelon tab/patch, Donepezil, Galantamine, Droxidopa, Clonazepam, Others	Yes, No	Categorical
Residential and environmental exposures history		
History of living near farm fields (within a half kilometer)	Yes, No, Don’t Know	Categorical
Main source of drinking water	City/town water supply; Bottled water; Spring water; Community well; Private well; Rainwater/cistern; River/lake/pond; Others	Categorical
History of working in plumbing, welding, or soldering	Yes, No, Don’t Know	Categorical
History of exposure heavy metals: Arsenic, Cadmium, Chromium, Copper, Lead, Mercury, Manganese, Nickel, or Zinc	Yes, No, Don’t Know	Categorical
History of mixing or applying pesticides: Herbicides, Fungicides, Insecticides, Fumigants, or other chemicals	Yes, No, Don’t Know	Categorical
Patient and physician perceptions		
Physician-perceived improvement in the patient’s condition since the last visit (Likert scale: 1 = Significant decline to 7 = Significant improvement)	Mean ± SD/Median ((nterquartile range (IQR))	Continuous
Patient-perceived improvement in their condition since the last visit (Likert scale: 1 = Significant decline to 7 = Significant improvement)	Mean ± SD/Median ((nterquartile range (IQR))	Continuous
Duration and frequency of care		
Duration of care at this clinic (months)	Mean ± SD/Median ((nterquartile range (IQR))	Continuous
Next follow-up appointment scheduled (months)	Mean ± SD/Median ((nterquartile range (IQR))	Continuous
Financial and health coverage		
Current health coverage status	Government funded, Private insurance, Self-paid, Family supported, Others	Categorical
Inability of the patient or their family to pay medical bills (last year)	Yes, No	Categorical
Level of patient agreement with the statement: “My illness has been a financial hardship for my family and me” (1 = Strongly disagree to 5 = Strongly agree)	Mean ± SD/Median ((nterquartile range (IQR))	Continuous
Level of patient confidence in their ability to control and manage most health problems (Likert scale: 0–10)	Mean ± SD/Median ((nterquartile range (IQR))	Continuous
Score for Financial Toxicity–Functional Assessment of Chronic Illness Therapy (COST-FACIT)	Mean ± SD/Median ((nterquartile range (IQR))	Continuous

Multilevel binary and ordinal logistic regression models will be employed to assess the relationships between previous histories of environmental exposures and the following PD-related outcome variables: rest tremors, dyskinesia, visual hallucinations, memory decline, freezing episodes, sleep disturbances, sleep behavior disorder, rapid eye movement sleep behavior disorder, Dopamine Dysregulation Syndrome, and PD symptom severity. The previous histories of environmental exposures to be explored include living near farm fields, the main source of drinking water, working in plumbing, welding, or soldering, exposure to heavy metals, mixing or applying pesticides, and drug use. Similarly, crude and adjusted ORs and 95% CIs will be reported and presented in a table as measures of these associations.

## Discussion

Over the last 25 years the prevalence of PD has risen in the MENASA region potentially as a result of aging population, sedentary lifestyle and expanding industrialization [14–16], however, prevalence of the disease in Asians and Blacks is reported to be 50% less as opposed to Whites [14, 16]. There is a need for accurate epidemiological data and to understand patients’ clinical characteristics and current practices in the region. Such data are crucial for assessing population needs, developing policies, and allocating healthcare and research resources. The urgent need for studies to understand PD related factors in this region has also been previously emphasized [14].

The primary aim of creating this consortium and database is to investigate the demographic and clinical attributes of patients with PD and the care provided for this population in the MENASA region. Large scale multicenter observational studies are important prerequisites for translational and interventional PD research in the MENASA region. Understanding the effect of PD on the overall wellbeing and daily functioning of affected individuals will provide the base for future follow-up pharmacological and non-pharmacological studies to manage these symptoms, facilitate early diagnosis, and improve quality of healthcare. Overall, the regional and ethnic disparities impact both the prevalence of PD and its clinical features. [14]. We suspect that the disease phenotype in MENASA region may be influenced by unique genetic, environmental and cultural factors. Our study may help identify additional risk factors that may not have been observed in Western countries yet. For instance, high rates of marriages between relatives in MENASA countries may be a risk factor for the region’s relatively high prevalence of genetic diseases [14, 17]. In many rural regions of MENASA countries, a large number of farmers are regularly exposed to various pesticides, which have been recognized as a contributing factor for development of PD [14, 15]. Although rural living has been cited as a potential factor for PD progression, a US based study found a higher rate and occurrence of PD in urban as compared to rural population [16]. The same study proposed debatable role of single toxin exposure such as pesticides or industrial toxins in development of PD [15]. On the contrary, the high prevalence of PD in Egyptian cities near the Nile Valley has been suggested to be attributable to industrial poisons draining into the Nile river [16]. Therefore, the current database can help identify cultural and environmental risk factors which may be unique to the MENASA region, as well as factors with are similar to those in western countries. Understanding these factors can improve the quality of care provided for these individuals and guide researchers in their interventional studies. Cultural variations such as lack of awareness about the disease, stigma regarding cognitive impairment and sexual dysfunction, and lack of health-related resources are few of the factors that we believe may rise in this study that may be considered unique to the MENASA region. We believe that some of the aforementioned cultural factors may directly influence data collection. Social stigma that is often present among different neurological conditions in the MENASA region may lead to symptom underreporting [17], especially symptoms such as depression and cognitive impairment. Hesitancy to participate in research has been previously reported in PD due to variability in healthcare access in the MENA region [18]. Religious beliefs, specifically those regarding destiny and illness may also influence the participation in research [19]. To overcome these possible factors, researchers in this study will collect data from various healthcare settings and recruit patients from different geographical and socioeconomic backgrounds. The researchers will also ensure anonymity and confidentiality of the collected data.

On the other hand, it is speculated that illiteracy in many MENASA countries may be the cause of delayed diagnosis, which in turn leads to undue delay in commencement of treatment and consequently poor wellbeing of PD patients [20]. There is a significant relationship between patients’ educational background, understanding the need for treatment, and consistency in compliance with medications and rehabilitation. Lack of availability of patient educational material and support groups in regional languages are other barriers that may contribute to poor patient understanding of disease and compliance to treatment [21]. Regional consensus showed significant need for developing educational programs for both patients and healthcare professionals [20]. Accessible high quality educational programs supported by the participation of professionals that provide care and support for PD patients are essential for better patients’ care, such as neurologists, researchers, PD nurses, and allied healthcare professionals. For example, supporting interested individuals to study in other countries under supervision, holding symposia directed toward delivering updates in the field, is anticipated to contribute to this regard.

This database will eventually provide real-world data that will help establish public health campaigns and initiatives to advocate PD patients, raising awareness and providing education. The database can also improve training and education for healthcare professionals through the establishment of continuing medical education (CME) programs providing healthcare workers with the best available evidence and updated practices in PD care.

Understanding regional PD treatment needs is also critical in prioritizing public health initiatives. Availability and affordability of PD treatment could be barriers to improving the quality of life of PD patients. Recent studies have shown the high economic burden of PD on the patient and the family in several MANASA regions [14, 15]. In many countries in the region, patient’s out-of-pocket cost is significantly high due to a lack of national healthcare coverage and private healthcare insurance coverage [20]. Furthermore, lack of access to device assisted therapies such as Deep Brain Stimulation (DBS) or infusion therapies may adversely affect the outcome of patients with advanced disease [20] Levodopa-carbidopa intestinal gel (LCIG) and continuous subcutaneous apomorphine infusion (CSAI) are examples of existing infusion therapies that are often used as an alternative option to oral medications in PD patients with inadequately controlled symptoms [20].

A multidisciplinary approach is the key to improving the quality of life of PD patients, given the complexity of the disease [22]. To help PD patients maintain maximum level of independence, physical rehabilitation therapies have been established as a supplement to medication use [5]. Reportedly limited availability of specialized physical, occupational, and speech rehabilitation contributes to adverse outcome of the disease [20, 21]. Deeply rooted sedentary lifestyle of most MENASA countries, lack of interest in exercise and absence of health insurance coverage may be barriers to rehabilitation. Besides this, women and older individuals of this region rely heavily on their families for transportation and access to rehabilitation services, making rehabilitation difficult to manage [21]. In addition to that, personalized care for PD patients is often achieved by professionals trained to provide support and deal with individual issues in PD. Parkinson’s Nurses (PN) are specialized to provide such care and have been shown to provide a pivotal role in the primary care of PD patients [15, 23]. In the MENASA region, a consensus statement by the MDS task force generated a priority need to have specialized professionals in movement disorders in this region [24]. This emphasizes the need to establish the PD database to facilitate the identification of gaps in the multidisciplinary care provided for PD patients in the MENASA region, to eventually help establish such needed interventions.

A significant limitation in the current multidisciplinary team providing care for PD patients in the MENASA region is the absence of PD nurse experts in the majority of participating centers. While professional nurses are essential in providing patient education, medication management, emotional support, and symptoms monitoring [25, 26]. This service is not available in most of the MENASA regions. This gap may lead to increased demands on neurologists, limited patient access to interventions on time, and discontinued disease management [27]. Identifying this limitation is crucial as it underlines the gap for potential improvement in the healthcare policy. Additionally, by adding data from centers with PD nurses to the database, future research may evaluate their impact on patient outcomes. A comparison between centers with and without specialized PD nurses could provide significant evidence of their important role in enhancing the quality of care for PD patients.

This database will provide an up-to-date status of the healthcare system provided for individuals with PD in the MENASA region. It will help understand region specific epidemiology, genetic predisposition, risk factors, clinical presentation, drug responsiveness, economic burden and quality of life of PD patients. Findings from this data can highlight the need to improve the quality of healthcare provided for these patients. Healthcare providers, researchers, and policymakers can utilize the findings from this study to improve the management options available for these individuals in the MENASA region and even globally in other underserved regions. This can be achieved by developing new therapeutic approaches and models of care for these individuals that are culturally and environmentally appropriate. Information from this database can help researchers identify environmental and genetic factors that are unique to the MENASA region. This will advance clinical research and lead to personalized treatment strategies. Healthcare providers can utilize patient data to capture the multidimensional PD symptom nature and eventually develop holistic care models. Furthermore, drug development can be enhanced by using the data to test new drugs, optimize existing treatments, and reduce adverse effects. Policymakers can generate recommendations and regulations that are evidence-based to prioritize the healthcare provided for these individuals as well as allocating funds to improve the resources available. For example, governments and policymakers can allocate resources effectively, establish financial aid programs, adjust insurance coverage, and ensure patients with PD have access to proper diagnostic tools, medications, and multidisciplinary care. Extending beyond the region, this database can eventually provide an opportunity for international researchers from global institutions to collaborate with MENASA institutions to exchange knowledge, develop new PD treatments, and foster global discussions.
