# Supplementary material for: Parkinson's Disease Database in the Middle East, North Africa, and South Asia Countries
Source: Int J Public Health. 2025 Jun 2;70:1608016. doi: 10.3389/ijph.2025.1608016 (PMC12264538; doi:10.3389/ijph.2025.1608016)
Supplement: Supplementary file 1 [file DataSheet1.docx]

| **Record identifier:** | | | | | | | | | | | **Date of visit:** _______________ DD/MM/YYYY | | | | | | | |
| --- | --- | --- | --- | --- | --- | --- | --- | --- | --- | --- | --- | --- | --- | --- | --- | --- | --- | --- |
| **Form completed by:**  **□** MD **□** RN **□** RA **□** Other | | | | | | **Clinician name:** _________________ | | | | | | | | **Institution name:** ___________________ | | | | |
| **Patient Data:** | | | | | | | | | | | | | | | | | | |
| **Case ID:** | | | | | | | | | | | | | | | | | | |
| **1. Country:**  _________________  **2. City:**  _________________ | | **3. DOB:**  _________________  (DD/MM/YYYY) | | | | | | | | | | | | **4. Sex:**   1. MALE 2. FEMALE | | | | |
|  |  | **5. Weight**: ______ KG | | | | | | | | | | | | **6. Height**: ___________M | | | | |
| **7. Race**   1. WHITE/CAUCASIAN 2. BLACK 3. BROWN 4. YELLOW 5. MULTI-RACIAL 6. OTHERS | | | | **8. Ethnicity:**   1. MIDDLE EASTERN ARAB 2. NORTH AFRICAN ARAB 3. BERBER 4. PERSIAN 5. KURDISH 6. ARMENIAN 7. TURKISH 8. BENGALI 9. PUNJABI 10. PASHTUN 11. SINDHI 12. BALOCHI 13. INDO-ARYAN 14. JEWS 15. AFRICAN (None-North-African) 16. NUBIANS 17. INDIAN 18. OTHERS | | | | | | | | | | | | | | |
| **Living situation:**  **9. Do you live with someone who can help you with activities of daily living if you need assistance?**   1. No 2. Yes   **9.1 If Yes:**   1. Family member or relative 2. Paid or other unrelated person | | | | | | | | | | | | | | | | | | |
| **10. During OPD visits, the patient is:**   1. UNACCOMPANIED 2. WITH REGULAR CARE PARTNER 3. WITH OTHER CAREGIVER 4. WITH OTHER PERSON | **11. Does the patient have a regular care partner?**   1. NO (If no, please skip the next question) 2. YES | | | | | | | | | | | | | **11.1 If yes:**  **Care partner employment**  **status**   1. EMPLOYED OUTSIDE OF HOME FULL TIME 2. EMPLOYED OUTSIDE OF HOME PART TIME 3. NOT EMPLOYED OUTSIDE OF HOME | | | | |
| **Level of education:**  **12.** TOTAL YEARS OF EDUCATION: ___________ YEARS  **12.1 Select what’s apply:**   1. LESS THAN HIGH SCHOOL GRADUATE 2. HIGH SCHOOL GRADUATE 3. SOME POST-HIGH SCHOOL EDUCATION OR ASSOCIATE’S DEGREE 4. BACHELOR’S DEGREE 5. GRADUATE DEGREE MASTER’S /PROFESSIONAL/ DOCTORAL) | | | | | | | | | | | | | | | | | | |
| **13. Marital status:**   1. SINGLE OR NEVER MARRIED 2. MARRIED OR DOMESTIC PARTNERSHIP 3. WIDOWED 4. DIVORCED OR SEPARATED | | | | | | | | | | | | **14. Current employment status:**   1. EMPLOYED OUTSIDE OF HOME FULL TIME 2. EMPLOYED OUTSIDE OF HOME PART TIME 3. NOT EMPLOYED OUTSIDE OF HOME | | | | | | |
| **Information about the disease** | | | | | | | | | | | | | | | | | | |
| **15. Year of 1^st^ onset of symptoms:**  _____________________ YYYY | | | | **16. Symptom at onset:**   1. MOTOR SYMPTOMS 2. NON-MOTOR SYMPTOM 3. BOTH | | | | | | | | | | **17. Side of symptom onset**:   1. RIGHT 2. LEFT 3. BILATERAL | | | | |
| **18. Year of PD diagnosis**: _____________________________ (YYYY) | | | | | | | | | | | | | | | | | | |
| **19. Can the patient stand unaided?**   1. NO 2. YES | | | | | | | | | | | | | | | | | | |
| **20. Can the patient engage in activities outside of home unaccompanied?**   1. NO 2. YES | | | | | | | | | | | | | | | | | | |
| **21. Does the patient attend a support group?**   1. NO 2. YES | | | | | | | | | | | | **21.1 If yes:**   1. IN PERSON 2. ONLINE 3. BOTH 4. OTHER | | | | | | |
| **Family History of PD** | | | | | | | | | | | | | | | | | | |
| **22. Does anyone else in the family have PD?**   1. NO 2. YES 3. DON’T KNOW | | | | | | | | | | **22.1 If Yes,**  **How many family members have been diagnosed with PD by a physician?**  **____________________** | | | | | | | | |
| **23.** Complete the boxes with the number of individuals, as appropriate. If there are none, please complete with 0 (zero):   \|  \| 1. **Affected** \| 1. **Unaffected** \| 1. **Don’t know** \| \| --- \| --- \| --- \| --- \| \| - 1. **Father** \|  \|  \|  \| \| - 1. **Mother** \|  \|  \|  \| \| - 1. **Sister(s)** \|  \|  \|  \| \| - 1. **Brother(s)** \|  \|  \|  \| \| - 1. **Child(ren)** \|  \|  \|  \| \| **Maternal side of family** \| \| \| \| \| - 1. **Grandparents** \|  \|  \|  \| \| - 1. **Aunt(s)** \|  \|  \|  \| \| - 1. **Uncle(s)** \|  \|  \|  \|  \| **Paternal side of family** \| \| \| \| \| --- \| --- \| --- \| --- \| \| **23.9 Grandparents** \|  \|  \|  \| \| **23.10 Aunt (s)** \|  \|  \|  \| \| **23.11 Uncle (s)** \|  \|  \|  \|   **Affected** = family member was diagnosed with PD by physician  **Unaffected** = family member shows no signs or symptoms suggestive of PD by physician  **Don't know** = subject doesn't have enough contact with family member to make judgement | | | | | | | | | | | | | | | | | | |
| **Environmental Exposures** | | | | | | | | | | | | | | | | | | |
| **Residential History** | | | | | | | | | | | | | | | | | | |
| **24. Have you ever lived near farm fields (within a half kilometer)?**   1. NO 2. YES 3. DON’T KNOW   **24.1** **If yes,** from year ___________________ to year _____________________  **25. What has been your main source of drinking water?**   1. CITY/TOWN WATER SUPPLY 2. BOTTLED WATER 3. SPRING WATER 4. COMMUNITY WELL 5. PRIVATE WELL 6. RAINWATER/CISTERN 7. RIVER/LAKE/POND 8. OTHER SOURCE OF WATER, SPECIFY_______________________________________ 9. DON’T KNOW   **26. Other than your main source of water, have you ever consumed any of the above-mentioned sources of water?**   1. NO 2. YES   **26.1 If yes, specify which** *_______________________________________*  **26.2 Age of consumption**: from *____________* to *_____________* years | | | | | | | | | | | | | | | | | | |
|  |  |  |  |  |  |  |  |  |  |  |  |  |  |  |  |  |  |  |
|  |  |  |  |  |  |  |  |  |  |  |  |  |  |  |  |  |  |  |
| **Head Injury** | | | | | | | | | | | | | | | | | | |
| **27. Have you ever suffered a head injury that affected your thinking, such as a concussion?**   1. NO (If no, please skip the next 2 questions) 2. YES (_________ times) 3. DON’T KNOW | | | | | | | | | | | | **27.1 If yes, when did you suffer head injury?**   1. -------- years BEFORE PD DIAGNOSIS 2. --------- years AFTER PD DIAGNOSIS  **27.2 Did you seek emergent/urgent care?** 3. NO 4. YES | | | | | | |
| **Heavy Metal Use**  (**For example:** **Arsenic, Cadmium, Chromium, Copper, Lead, Mercury, Manganese, Nickel, Zinc**) | | | | | | | | | | | | | | | | | | |
| **28. Have you worked in plumbing, welding or soldering, … or had other exposure to metals such as [Arsenic, Cadmium, Chromium, Copper, Lead, Mercury, Manganese, Nickel, Zinc]?**   1. NO (If NO, skip the next 2 questions) 2. YES 3. DON’T KNOW | | | | | | | **28.1 If YES: which work or metal?**  _____________________  ____________________  ____________________ | | | | | | | | | **28.2 Age at heavy metal usage:**  From age _*_____*to*_____* years | | |
| **Pesticides Use** | | | | | | | | | | | | | | | | | | |
| **29. Over your lifetime, have you ever mixed or applied any type of pesticide, including herbicides (to kill weeds), fungicides (to kill fungus/mold), insecticides (to kill insects), rodenticides (to kill rats/mice), or fumigants (gas used to kill fungus/mold or insects) or ever use chemicals to kill pests, plants,**  **weeds, or mildew?**   1. NO (If NO, skip the next 4 questions from) 2. YES 3. DON’T KNOW | | | | | | | **29.1 If YES, which work or Pesticides?**  _____________________  ___________________ | | | | | | | | | **29.2 Age at Pesticides usage:**  From age _*_____*to*_____* years | | |
| **29.3 How many years in total did you have to mix or apply pesticides?**   1. _____________YEARS 2. DON’T KNOW | | | | | | | | | | | | **29.4 During these years, about how many days per year did you use pesticides?**   1. 1-5 DAYS 2. 6-10 DAYS 3. 11-30 DAYS 4. MORE THAN 30 DAYS 5. DON’T KNOW | | | | | | |
| **Tobacco** | | | | | | | | | | | | | | | | | | |
| **30. Do you smoke or have you ever smoked cigarettes?**   1. NO (If NO, skip the next question) 2. YE | | | | | | **30.1 If Yes,**  **How many years?**  **How many packets/ day?**  **Please check all that apply**   1. Less than ½ pack per day for_______ years. 2. Equal to or more than ½ pack but less than 1 pack per day for________ years. 3. Equal to or more than 1 pack but less than 2 pack per day for ________ years. 4. Equal to or more than 2 packs per day for________ years. | | | | | | | | | | | | |
| **31. Any form of tobacco use other than smoking?**   1. NO (If no, please skip the next question) 2. YES | | | | | | | | | | | | **31.1 If yes, select from:**   1. CHEWABLE TOBACCO 2. HUQQA 3. SHEESHA 4. OTHER FORMS: specify ________________ | | | | | | |
| **Caffeine** | | | | | | | | | | | | | | | | | | |
| **32. Do you drink coffee or tea? 32.1 If yes, What type?**   1. NO **a.** Coffee 2. YES **b.** Tea   **c.** Both  **32.2 If Yes, mark specific type(s) of coffee/Tea?**   1. Latte/Cappuccino (coffee with milk) 2. Americano/Espresso 3. Arabic coffee 4. Black tea 5. Green tea 6. Black tea with milk 7. Any other, please specify ______________________________   **32.3 Check all that apply (Fill the first blank from (a-g) choices from Question 32.2)**   1. Less than 2 cups of _________ a week for____ years. 2. 2-6 cups of _________ a week for __________ years. 3. 1-2 cups of _________ a day for____________ years. 4. 3-5 cups of _________ a day for____________ years. 5. 6 or more cups of _________ a day for_______ years. | | | | | | | | | | | | | | | | | | |
| **33.** How much caffeinated **soda** do you (or did you) drink and for how many years?  (A can of soda is 12 oz.)   1. No 2. Yes   **33.1 If Yes,**  **Check all that apply**   1. Less than 1 can a week for_____ years. 2. 2-3 cans a week for __________ years. 3. 1-2 cans a day for____________ years. 4. 3-5 cans a day for____________ years. 5. 6 or more cans a day for_______ years. | | | | | | | | | | | | | | | | | | |
| **Drug Abuse** | | | | | | | | | | | | | | | | | | |
| **34. During your lifetime, did you ever use chemicals or drugs as amphetamine, methamphetamine, cocaine, or heroin?**   1. NO 2. YES 3. PREFER NOT TO ANSWER   **34.1 If YES,** please fulfill the following table:   \| **34.1 a**  **Drug name** \| **34.1 b Started at age of** \| **34.1 c**  **Stopped at age of** \| **34.1 d**  **Still using (Yes/No)** \| **34.1 e**  **frequency per month** \| \| --- \| --- \| --- \| --- \| --- \| \|  \|  \|  \|  \|  \| \|  \|  \|  \|  \|  \| \|  \|  \|  \|  \|  \| \|  \|  \|  \|  \|  \| \|  \|  \|  \|  \|  \| \|  \|  \|  \|  \|  \| | | | | | | | | | | | | | | | | | | |
| **Clinical characteristics of disease** | | | | | | | | | | | | | | | | | | |
| **35. Ever experienced rest tremors since diagnosis of PD:**   1. NO 2. YES | | | | | | | | | | | | | | | | | | |
| **36. Dyskinesia over last 30 days:**   1. NO 2. YES | | | | | | | | | | - 1. **Does dyskinesia limit activities?**  1. NO 2. YES | | | | | | | | |
| **37. Does pt. have wearing-off periods in the last 30 days?**   1. NO (If no, please skip the next 2 questions) 2. YES | | | | | | | | **37.1 If yes,**  **medication effect at this visit?**   1. NO 2. YES 3. IN BETWEEN | | | | | | | | | **37.2 Do they limit patient activities?**   1. NO 2. YES | |
| **38. Hoehn & Yahr stage at this visit:**   1. Stage 0: No signs of disease 2. Stage 1.0: Symptoms are very mild; unilateral involvement only 3. Stage 1.5: Unilateral and axial involvement 4. Stage 2: Bilateral involvement without impairment of balance 5. Stage 2.5: Mild bilateral disease with recovery on pull test 6. Stage 3: Mild to moderate bilateral disease; some postural instability; physically independent 7. Stage 4: Severe disability; still able to walk or stand unassisted 8. Stage 5: Wheelchair bound or bedridden unless aided | | | | | | | | | | | | | | | | | | |
| **39. Dopamine dysregulation syndrome**   1. NO 2. YES | | | | | | | | | | | | | | | | | | |
| **40. Non-motor symptoms if present at the onset: (Please select all that apply)**   1. DEPRESSION 2. ANXIETY 3. MILD COGNITIVE IMPAIRMENT 4. APATHY 5. FATIGUE 6. INSOMNIA 7. RLS 8. REM DISORDERS 9. DAYTIME SLEEPINESS 10. CONSTIPATION 11. URINARY TRACT SYMPTOMS 12. PAIN / MUSCLE CRAMPS 13. ORTHOSTATIC HYPOTENSION/ DIZZINESS WHEN STANDING 14. ANOSMIA | | | | | | | | | | | | | | | | | | |
| **Clinical Impression** | | | | | | | | | | | | | | | | | | |
| **41. Frequency of falls (last 3 months)**   1. NONE 2. RARE 3. MONTHLY 4. WEEKLY 5. DAILY | | | | | | | | | | | | | | | | | | |
| **42. Is the patient having visual hallucinations?**   1. NO (If no, please skip the next 2 questions) 2. YES | | | | | | | | | **42.1 If Yes, when did hallucinations start?**   1. BEFORE PD DIAGNOSIS 2. AFTER PD DIAGNOSIS   **42.1.a If the answer is “AFTER PD DIAGNOSIS”, please specify**   1. AT TIME OF DIAGNOSIS 2. 1-5 YEARS OF DIAGNOSIS 3. 6-10 YEARS OF DIAGNOSIS 4. >10 YEARS OF DIAGNOSIS | | | | | | | | | |
| **43. Is the patient having memory decline?**   1. NO (If no, please skip the next 2 questions) 2. YES | | | | | | | | | **43.1 When did memory decline start?**   1. BEFORE PD DIAGNOSIS 2. AFTER PD DIAGNOSIS   **43.1.a If the answer is “AFTER PD DIAGNOSIS”, please specify**   1. AT TIME OF DIAGNOSIS 2. 1-5 YEARS OF DIAGNOSIS 3. 6-10 YEARS OF DIAGNOSIS 4. >10 YEARS OF DIAGNOSIS | | | | | | | | | |
| **44. Is the patient having freezing episodes?**   1. NO (If no, please skip the next 2 questions) 2. YES | | | | | **44.1 When did the patient start freezing?**   1. BEFORE PD DIAGNOSIS 2. AFTER PD DIAGNOSIS   **44.1.a If the answer is “AFTER PD DIAGNOSIS”, please specify**   1. AT TIME OF DIAGNOSIS 2. 1-5 YEARS OF DIAGNOSIS 3. 6-10 YEARS OF DIAGNOSIS 4. >10 YEARS OF DIAGNOSIS | | | | | | | | | | | | **44.2 Is patient incapacitated by freezing?**   1. NO 2. YES | |
| **Sleep Issues:** | | | | | | | | | | | | | | | | | | |
| **45. Is the patient having sleep disturbances?**   1. NO (If no, please skip the next 5 questions) 2. YES | | | | | **45.1 If Yes,**  **When did the sleep disturbance start?**   1. BEFORE PD DIAGNOSIS 2. AFTER PD DIAGNOSIS   **45.2 Is patient incapacitated by sleep disturbance**   1. NO 2. YES | | | | | | | | | | | | | |
| **45.3 What type of sleep disorder does the patient have?**   1. RBD 2. RLS 3. Daytime sleepiness 4. Insomnia | | | **45.4 How often does the patient get sleep problems?**  ________________DAYS/WEEK | | | | | | | | | | | | **45.5 Is the patient taking any medicine for sleep disorder?**   1. NO 2. YES   **45.5.a If yes,**  **Is the medicine helping sleep disorders effectively?**   1. NO 2. YES | | | |
| **46. Screening Question for RBD:**  **“Have you ever been told, or suspected yourself, that you seem to ‘act out your dreams’ while asleep (for example, punching, flailing your arms in the air, making running movements, etc.)?**   1. NO 2. YES | | | | | | | | | | | | | | | | | | |
| **47. Comorbid Conditions:** | | | | | | | | | | | | | | | | | | |
| **PROBLEM** | | | | | | | **DO YOU HAVE THE PROBLEM?**   1. NO 2. YES | | | | | | **DO YOU RECEIVE TREATMENT FOR IT?**   1. NO 2. YES | | | | | **DOES IT LIMIT YOUR ACTIVITIES?**   1. NO 2. YES |
| **47.1 High blood pressure** | | | | | | |  | | | | | |  | | | | |  |
| **47.2 Heart disease** | | | | | | |  | | | | | |  | | | | |  |
| **47.3 Anemia** | | | | | | |  | | | | | |  | | | | |  |
| **47.4 Lung disease** | | | | | | |  | | | | | |  | | | | |  |
| **47.5 Cancer** | | | | | | |  | | | | | |  | | | | |  |
| **47.6 Diabetes** | | | | | | |  | | | | | |  | | | | |  |
| **47.7 Ulcer or stomach disease** | | | | | | |  | | | | | |  | | | | |  |
| **47.8 Constipation** | | | | | | |  | | | | | |  | | | | |  |
| **47.9 Liver disease** | | | | | | |  | | | | | |  | | | | |  |
| **47.10 Kidney disease** | | | | | | |  | | | | | |  | | | | |  |
| **47.11 Depression** | | | | | | |  | | | | | |  | | | | |  |
| **47.12 Psychosis** | | | | | | |  | | | | | |  | | | | |  |
| **47.13 Osteoarthritis, degenerative arthritis** | | | | | | |  | | | | | |  | | | | |  |
| **47.14 Back pain** | | | | | | |  | | | | | |  | | | | |  |
| **47.15 Rheumatoid arthritis** | | | | | | |  | | | | | |  | | | | |  |
| **47.16 BPH** | | | | | | |  | | | | | |  | | | | |  |
| **47.17 Other medical problems**  (Please write in) | | | | | | |  | | | | | |  | | | | |  |
| **48. ER Visits (last 12 months)**   1. NO 2. YES ( # COUNT ____________) | | | | | | | | | | | | | | | | | | |
| **49. Hospital Admissions (last 12 months)**   1. NO 2. YES ( # COUNT ____________) | | | | | | | | | | | | | | | | | | |
| **If yes**, reason for **admissions (last 12 months):** | | | | | | | | | | | | | | | | | | |

| **49.1 Any injury or trauma?**   1. NO (If no, please skip the next 2 questions) 2. YES | | **49.1.a If Yes,**  **Hip fracture?**   1. NO 2. YES | | | | | **49.1.b** Fracture other than hip?   1. NO 2. YES | | |
| --- | --- | --- | --- | --- | --- | --- | --- | --- | --- |
| **49.2 Any infection?**   1. NO (If no, please skip the next 2 questions) 2. YES | **49.2.a If Yes, Pneumonia?**   1. NO 2. YES | | | | | **49.2.b** Infection other than pneumonia?   1. NO 2. YES | | | |
| **49.3 Any behavioral/ mental health diagnosis?**   1. NO (If no, please skip the next 3 questions) 2. YES | | | **If Yes** | | | | | | |
|  |  |  | **49.3.a Confusion?**   1. NO 2. YES | | | | | | |
| **49.3.b Psychosis?**   1. NO 2. YES | | | **49.3.c Mood disorder?**   1. NO 2. YES | | | | | | |
| **49.4 Gastrointestinal issues**   1. NO (If no, please skip the next question) 2. YES | | | **49.4.a If Yes**  Specify: __________________________ | | | | | | |
| **49.5 DBS-related**   1. NO (If no, please skip the next question) 2. YES | | | **49.5.a If Yes**  Specify: __________________________ | | | | | | |
| **49.6 Other**   1. NO (If no, please skip the next question) 2. YES | | | **49.6.a If Yes**  Specify: ________________________ | | | | | | |
| **Medications** | | | | | | | | | |
| **50. Compliance with PD medications**   1. NOT COMPLIANT 2. COMPLIANT | | | | | | | | | |
| **51. PD Medications started at time of diagnosis:**   1. NO 2. YES   51.1. If the answer in NO, after how many months/years were medications started?  ____________________ months / years | | | | | | | | | |
| **Medications (taken daily)** | | | | | | | | | |
| **52. Patient’s PD Related Medications:** | | | | | | | | | |
| **Medication Name** | | | | | **Dose** | | | | **Frequency** |
| **52.1 Amantadine:**   1. NO 2. YES 3. NOT AVAILABLE | | | | | **52.1.a**   1. 100MG CAPSULE 2. 100MG TABLET | | | | **52.1.b**  Number of tablets/ number of times a day: |
| **52.2 Trihexyphenidyl:**  **Procyclidine Hydrochloride**   1. NO 2. YES 3. NOT AVAILABLE | | | | | **52.2.a**  2MG  5MG | | | | **52.2.b** Number of tablets/ number of times a day: |
| **52.3 Carbidopa/levodopa-Immediate release:**   1. NO 2. YES 3. NOT AVAILABLE | | | | | **52.3.a**  10/100  25/100  &  25/250 | | | | **52.3.b**  Number of tablets/ number of times a day: |
| **52.4 Carbidopa/levodopa-controlled release:**   1. NO 2. YES 3. NOT AVAILABLE | | | | | **52.4.a**  25/100  50/200 | | | | **52.4.b**  Number of tablets/ number of times a day: |
| **52.5 Entacapone (Comtan):**   1. NO 2. YES 3. NOT AVAILABLE | | | | | **52.5.a**  200 | | | | **52.5.b**  No of tablets/no of times in a day: |
| **52.6 Carbidopa/levodopa-extended release capsules (Rytary):**   1. NO 2. YES 3. NOT AVAILABLE | | | | | **52.6.a**  23.7/95  36.25/145  48.75/195  61.25/245 | | | | **52.6.b**  Number of capsules/number of times a day: |
| **52.7 Carbidopa/levodopa/entacapone (Stalevo):**   1. NO 2. YES 3. NOT AVAILABLE | | | | | **52.7.a**  12.5/50/200  18.75/75/200  25/100/200  31.25/125/200  37.5/150/200  50/200/200 | | | | **52.7.b**  Number of tablets/number of times a day: |
| **52.8 Levodopa inhalation powder (Inbrija):**   1. NO 2. YES 3. NOT AVAILABLE | | | | | **52.8.a** 42MG | | | | **52.8.b**  Number of times per day: |
| **52.9 Selegiline:**   1. NO 2. YES 3. NOT AVAILABLE | | | | | **52.9.a**  5MG (TABLET)  5MG (CAPSULE) | | | | **52.9.b**  Number of tablets/number of times a day: |
| **52.10 Safinamide:**   1. NO 2. YES 3. NOT AVAILABLE | | | | | **52.10.a**  50MG  100MG | | | | **52.10.b**  Number of tablets/number of times a day: |
| **52.11 Rasagiline:**   1. NO 2. YES 3. NOT AVAILABLE | | | | | **52.11.a**  0.5MG  1MG | | | | **52.11.b**  Number of times a day: |
| **52.12 Pramipexole Immediate release:**   1. NO 2. YES 3. NOT AVAILABLE | | | | | **52.12.a**  DOSE/DAY: | | | | **52.12.b**  Number of tablets/number of times a day: |
| **52.13 Pramipexole ER:**   1. NO 2. YES 3. NOT AVAILABLE | | | | | **52.13.a**  DOSE/DAY: | | | | **52.13.b**  Number of times a day: |
| **52.14 Ropinirole-immediate release:**   1. NO 2. YES 3. NOT AVAILABLE | | | | | **52.14.a**  DOSE: | | | | **52.14.b**  Number of tablets/number of times a day: |
| **52.15 Ropinirole-ER:**   1. NO 2. YES 3. NOT AVAILABLE | | | | | **52.15.a**  2MG  4MG  6MG  8MG  12MG | | | | **52.15.b**  Number of times a day: |
| **52.16 Rotigotine transdermal patch:**   1. NO 2. YES 3. NOT AVAILABLE | | | | | **52.16.a**  1MG  2MG  3MG  4MG  6MG  8MG | | | | **52.16.b**  Number of times a day: |
| **52.17 Apomorphine SL wafer (Kynmobi):**   1. NO 2. YES 3. NOT AVAILABLE | | | | | **52.17.a**  TIMES USED/WEEK IN LAST 3 MONTHS: | | | | **52.17.b**  Number of films/number of times a day: |
| **52.18 Apomorphine injection:**   1. NO 2. YES 3. NOT AVAILABLE | | | | | **52.18.a**  10MG  TIMES/WEEK IN LAST 3 MONTHS: | | | | **52.18.b**  Number of times a day: |
| **52.19 Bromocriptine:**   1. NO 2. YES 3. NOT AVAILABLE | | | | | **52.19.a**  5MG CAPSULE  2.5MG TABLET  DOSE: | | | | **52.19.b**  Number of tablets /number of times a day: |
| **52.20 Istradefylline (Nourianz):**   1. NO 2. YES 3. NOT AVAILABLE | | | | | **52.20.a**  DOSE: | | | | **52.20.b**  Number of tablets /number of times a day: |
| **52.21 Quetiapine:**   1. NO 2. YES 3. NOT AVAILABLE | | | | | **52.21.a**  DOSE: | | | | **52.21.b**  Number of tablets /number of times a day: |
| **52.22 Quetiapine ER:**   1. NO 2. YES 3. NOT AVAILABLE | | | | | **52.22.a**  DOSE: | | | | **52.22.b**  Number of tablets /number of times a day: |
| **52.23 Clozapine:**   1. NO 2. YES 3. NOT AVAILABLE | | | | | **52.23.a**  DOSE: | | | | **52.23.b**  Number of tablets /number of times a day: |
| **52.24 Pimavanserin:**   1. NO 2. YES 3. NOT AVAILABLE | | | | | **52.24.a**  DOSE: | | | | **52.24.b**  Number of tablets /number of times a day: |
| **52.25 Rivastigmine:**  **Exelon tab/patch**   1. NO 2. YES 3. NOT AVAILABLE | | | | | **52.25.a**  DOSE: | | | | **52.25.b**  Number of times a day: |
| **52.26 Donepezil:**   1. NO 2. YES 3. NOT AVAILABLE | | | | | **52.26.a**  DOSE: | | | | **52.26.b**  Number of tablets /number of times a day: |
| **52.27 Galantamine(Reminyl):**   1. NO 2. YES 3. NOT AVAILABLE | | | | | **52.27.a**  4 MG  8 MG | | | | **52.27.b**  Number of tablets /number of times a day: |
| **52.28 Droxidopa (NORTHERA®):**   1. NO 2. YES 3. NOT AVAILABLE | | | | | **52.28.a**  DOSE: | | | | **52.28.b**  Number of tablets /number of times a day: |
| **52.29 Clonazepam:**   1. NO 2. YES 3. NOT AVAILABLE | | | | | **52.29.a**  DOSE: | | | | **52.29.b**  Number of tablets /number of times a day: |
| **Other Medications Taken** (Please write in)  Other anti PD or medications for other chronic illness | | | | | | | | | |
| **52.30 Medication Name** | | | | **52.30.a Dose** | | | | **52.30.b Frequency** | |
| 1. | | | |  | | | |  | |
| 2. | | | |  | | | |  | |
| 3. | | | |  | | | |  | |
| 4. | | | |  | | | |  | |
| 5. | | | |  | | | |  | |
| 6. | | | |  | | | |  | |
| 7. | | | |  | | | |  | |
| 8. | | | |  | | | |  | |
| 9. | | | |  | | | |  | |
| 10. | | | |  | | | |  | |

| **53. Other Therapies:** | | | | | |
| --- | --- | --- | --- | --- | --- |
| **Therapy Type** | | **BEFORE VISIT (Current Treatment)**   1. NOT USED 2. YES, IN THE LAST 3 MONTHS 3. YES, 4–12 MONTHS AGO | | | **AFTER VISIT**  **(New / Continued)**   1. To be started 2. To be continued 3. Stopped/not needed |
| **53.1 Physical therapist** | |  | | |  |
| **53.2 Occupational therapist** | |  | | |  |
| **53.3 SLP for communication** | |  | | |  |
| **53.4 SLP for swallowing** | |  | | |  |
| **53.5 Dietitian** | |  | | |  |
| **54. Mental HEALTH** | | | | | |
| **54.1 Psychologist** | |  | | |  |
| **54.2 Psychiatrist** | |  | | |  |
| **55. Surgical treatment** | | | | | |
| **55. Surgical treatment**   1. No (If no, please skip the next 4 questions) 2. Yes   **55.1 If yes: which type of surgery?**   1. Deep Brain Stimulation (DBS) 2. Lesion | **55.2 If Deep brain stimulation (DBS), Is it:**   1. UNILATERAL (Right side of the brain or Left side 2. BILATERAL   **55.4** DBS surgery was done _______________ years ago | | | **55.3 Targets**:   1. STN 2. PPN 3. GPI 4. OTHER | |
| **56. Infusion pump inserted?**   1. NO 2. YES   **56.1** If **yes, which type of infusion?**   1. LCIG PUMP 2. APOMORPHINE PUMP   **56.2** If **yes,** was done _______________ years ago | | | | | |
| **57. Exercise program (at present)** | | | 1. NO PROGRAM (0 HOURS) 2. NUMBER OF HOURS PER WEEK OF EXERCISE ____________ | | |
| **INTENSITY/EXAMPLES** | | | **FREQUENCY** | | |
| 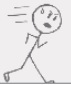  **57.1 How many hours per week do you do vigorous exercise?**   - Stair climbing for exercise - Swimming laps - Weight lifting | | | **57.1.a**  NUMBER OF HOURS/WEEK __________  write 0 IF NONE | | |
| 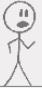  **57.2 How many hours per week do you do MODERATE exercise?**   - Fast/brisk walking pace or walking on hills - Dancing Tai chi, yoga, - Pilates Arm or leg cycling - Pool aerobics | | | **57.2.a**  NUMBER OF HOURS/WEEK __________  write 0 IF NONE | | |
| 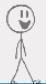  **57.3 How many hours per week do you do LIGHT exercise or activities?**   - Walking at a usual or leisurely pace - Chores in house or yard - Seated exercise routine | | | **57.3.a**  NUMBER OF HOURS/WEEK __________  write 0 IF NONE | | |
| **Current Clinical Status** | | | | | |
| **58. When was the patient last seen in the clinic?**  ____________ Months ago  **59. On a 7 point scale, with 4 being “unchanged,” how would you rate the patient’s current status versus last visit?**  **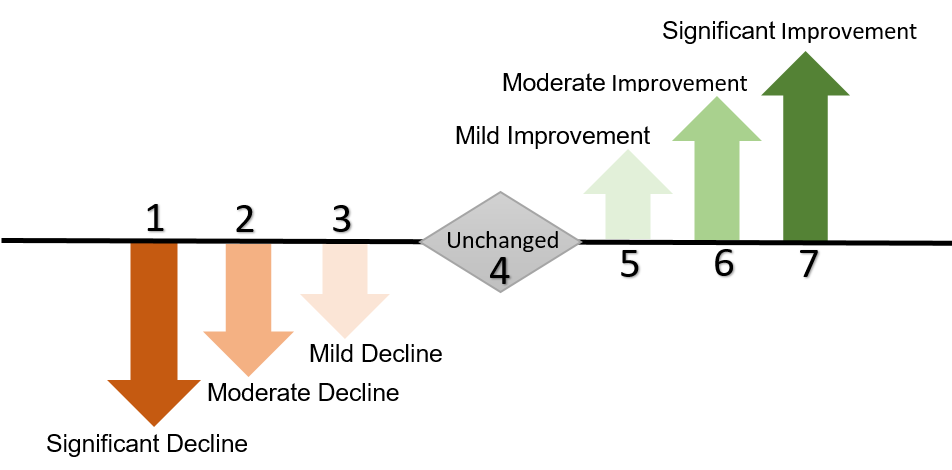**  **60. On a 7 point scale, with 4 being “unchanged,” how would the patient rate his/her current status versus last visit?**  **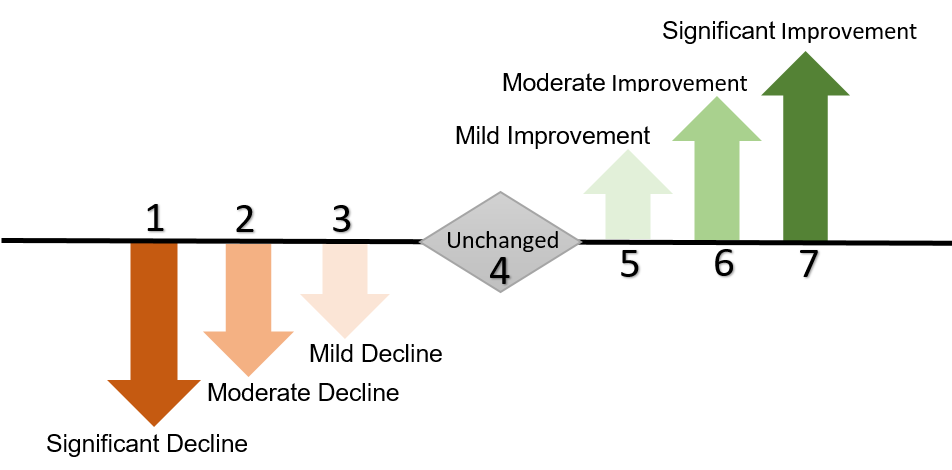** | | | | | |
| **Duration and Frequency of Care:** | | | | | |
| **61. For how long have you been receiving care at this clinic?** | | | _____________________________ YEARS  ___________________________MONTHS | | |
| **62. After how long is the next follow-up appointment scheduled?** | | | ___________________________MONTHS | | |
| **Health Cost** | | | | | |
| **63. Please choose your current health coverage status**   - 1. Government funded   2. Private insurance   3. Self-paid   4. Family supported   5. Others   **64. In the past 12 months, do you or anyone in your family/household have any medical bills that you are unable to pay?)**   1. NO 2. YES 3. PREFER NOT TO ANSWER 4. DON’T KNOW | | | | | |
| **65. My illness has been a financial hardship to my family and me**  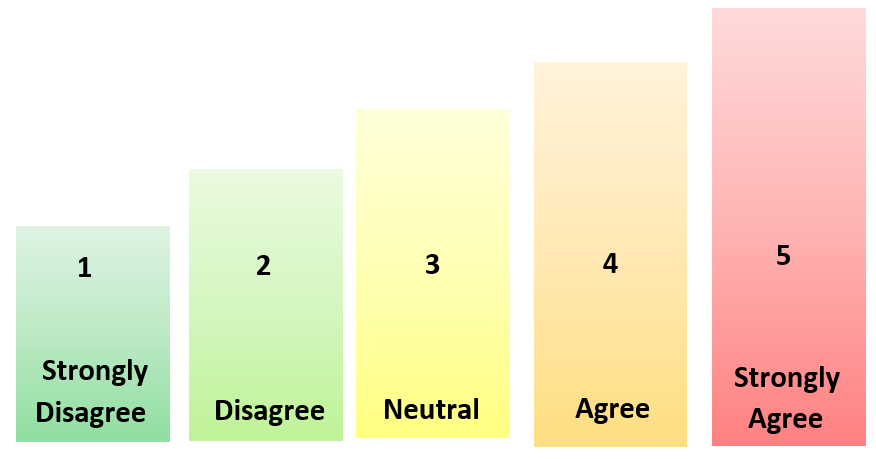 | | | | | |
| **66. How confident are you that you can control and manage most of your health problems?**  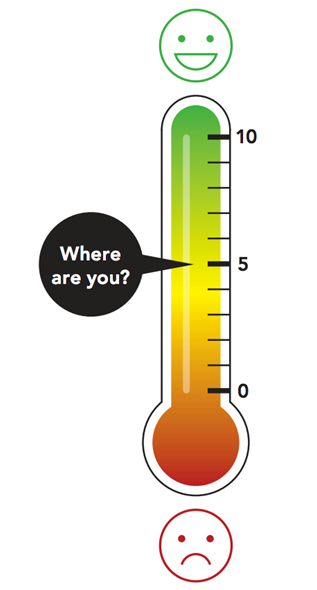 | | | | | |
| **COST – FACIT (Version 2)**  **67. Below is a list of statements that other people with your illness have said are important. Please circle or mark one answer per line to indicate your response as it applies in the past 3 months**   \|  \| \| **a**  **Not at all** \| **b**  **A little bit** \| **c**  **Somewhat** \| **d**  **Quite a bit** \| **f**  **Very much** \| \| --- \| --- \| --- \| --- \| --- \| --- \| --- \| \| **67.1** \| **I know that I have enough money in savings, retirement, or assets to cover the costs of my treatment** \|  \|  \|  \|  \|  \| \| **67.2** \| **My out-of-pocket medical expenses are more than I thought they would be** \|  \|  \|  \|  \|  \| \| **67.3** \| **I worry about the financial problems I will have in the future as a result of my illness or treatment** \|  \|  \|  \|  \|  \| \| **67.4** \| **I feel I have no choice about the amount of money I spend on care** \|  \|  \|  \|  \|  \| \| **67.5** \| **I am frustrated that I cannot work or contribute as much as I usually do** \|  \|  \|  \|  \|  \| \| **67.6** \| **I am satisfied with my current financial situation** \|  \|  \|  \|  \|  \| \| **67.7** \| **I am able to meet my monthly expenses** \|  \|  \|  \|  \|  \| \| **67.8** \| **I feel financially stressed** \|  \|  \|  \|  \|  \| \| **67.9** \| **I am concerned about keeping my job and income, including work at home** \|  \|  \|  \|  \|  \| \| **67.10** \| **My disease or treatment has reduced my satisfaction with my present financial situation** \|  \|  \|  \|  \|  \| \| **67.11** \| **I feel in control of my financial situation** \|  \|  \|  \|  \|  \| \| **67.12** \| **My illness has been a financial hardship to my family and me** \|  \|  \|  \|  \|  \| | | | | | |
| **(68) Comments** | | | | | |
|  | | | | | |
